# Circulating miRNAs in Untreated Breast Cancer: An Exploratory Multimodality Morpho-Functional Study

**DOI:** 10.3390/cancers11060876

**Published:** 2019-06-22

**Authors:** Mariarosaria Incoronato, Anna Maria Grimaldi, Peppino Mirabelli, Carlo Cavaliere, Chiara Anna Parente, Monica Franzese, Stefania Staibano, Gennaro Ilardi, Daniela Russo, Andrea Soricelli, Onofrio Antonio Catalano, Marco Salvatore

**Affiliations:** 1 IRCCS SDN, 80143 Naples, Italy; agrimaldi@sdn-napoli.it (A.M.G.); pmirabelli@sdn-napoli.it (P.M.); ccavaliere@sdn-napoli.it (C.C.); caparente@sdn-napoli.it (C.A.P.); mfranzese@sdn-napoli.it (M.F.); soricelli@uniparthenope.it (A.S.); direzionescientifica@sdn-napoli.it (M.S.); 2 Department of Advanced Biomedical Sciences, Federico II University, 80131 Naples, Italy; staibano@unina.it (S.S.); gennaro.ilardi@gmail.com (G.I.); danielarusso83@yahoo.it (D.R.); 3 Department of Motor Sciences & Wellness, University of Naples Parthenope, 80133 Naples, Italy; 4 Department of Radiology, Athinoula A. Martinos Center for Biomedical Imaging, Massachusetts General Hospital, Harvard Medical School, Charlestown, MA 02129, USA

**Keywords:** circulating miRNAs, breast cancer, imaging parameters, PET/MRI, biomarkers

## Abstract

The aim of this study was to identify new disease-related circulating miRNAs with high diagnostic accuracy for breast cancer (BC) and to correlate their deregulation with the morpho-functional characteristics of the tumour, as assessed in vivo by positron emission tomography/magnetic resonance (PET/MR) imaging. A total of 77 untreated female BC patients underwent same-day PET/MR and blood collection, and 78 healthy donors were recruited as negative controls. The expression profile of 84 human miRNAs was screened by using miRNA PCR arrays and validated by real-time PCR. The validated miRNAs were correlated with the quantitative imaging parameters extracted from the primary BC samples. Circulating miR-125b-5p and miR-143-3p were upregulated in BC plasma and able to discriminate BC patients from healthy subjects (miR-125-5p area under the receiver operating characteristic ROC curve (AUC) = 0.85 and miR-143-3p AUC = 0.80). Circulating CA15-3, a soluble form of the transmembrane glycoprotein Mucin 1 (MUC-1) that is upregulated in epithelial cancer cells of different origins, was combined with miR-125b-5p and improved the diagnostic accuracy from 70% (CA15-3 alone) to 89% (CA15-3 plus miR-125b-5p). MiR-143-3p showed a strong and significant correlation with the stage of the disease, apparent diffusion coefficient (ADC_mean_), reverse efflux volume transfer constant (Kep_mean_) and maximum standardized uptake value (SUV_max_), and it might represent a biomarker of tumour aggressiveness. Similarly, miR-125b-5p was correlated with stage and grade 2 but inversely correlated with the forward volume transfer constant (Ktrans_mean_) and proliferation index (Ki67), suggesting a potential role as a biomarker of a relatively more favourable prognosis. In situ hybridization (ISH) experiments revealed that miR-143-3p was expressed in endothelial tumour cells, miR-125-5p in cancer-associated fibroblasts, and neither in epithelial tumour cells. Our results suggested that miR-125-5p and miR-143-3p are potential biomarkers for the risk stratification of BC, and Kaplan-Maier plots confirmed this hypothesis. In addition, the combined use of miR-125-b-5p and CA15-3 enhanced the diagnostic accuracy up to 89%. This is the first study that correlates circulating miRNAs with in vivo quantified tumour biology through PET/MR biomarkers. This integration elucidates the link between the plasmatic increase in these two potential circulating biomarkers and the biology of untreated BC. In conclusion, while miR-143-3b and miR-125b-5p provide valuable information for prognosis, a combination of miR-125b-5p with the tumour marker CA15-3 improves sensitivity for BC detection, which warrants consideration by further validation studies.

## 1. Introduction

According to GLOBOCAN 2018, a project of the International Agency for Research on Cancer (IARC), approximately 2.1 million women (1.7 million in 2012) were diagnosed with breast cancer (BC) worldwide, and there were approximately 6.9 million women (6.3 million in 2012) who had been diagnosed with BC in the previous five years [1]. BC is also the most common cause of cancer death among women, with 627,000 deaths in 2018. Early diagnosis plays a major role in fighting BC. However, on a worldwide scale, breast imaging techniques such as screening mammography and ultrasound have intrinsic limitations related to economic costs, limited technology availability in underdeveloped countries, the necessity of well-trained radiologists, and an overall unsatisfactory performance regarding the images. Screening mammography performance indexes vary considerably for sensitivity and specificity, influencing the ability of mammography to reach its full potential for decreasing BC mortality [2]; moreover, ultrasound plus mammography can indicate cancers in high-risk women, but the number of false positives are increased [3].

Circulating biomarkers are biomolecules released into the bloodstream by both tumour cells and other types of neighbouring cells [4]. Their quantification and identification are considered an economical, non-invasive diagnostic method that provides information on the presence and/or absence of the disease as well as its evolution. To date, the carcinoembryonic antigen (CEA) [5] and the soluble form of Mucin 1 (MUC-1) protein (CA15-3) [6] are the most commonly used serum biomarkers for the clinical monitoring of BC patients. Although these biomarkers have been recognized from the international healthcare system as molecules used for the management of BC patients, current guidelines suggest that they fail in the early diagnosis of the disease, having a clinical role only during follow-up [7]. Therefore, there is a compelling need to discover potential biomarkers that might be useful for diagnosing BC. The ideal tumour marker should have high specificity, sensitivity and predictive value and be detectable by an accurate, rapid, simple and inexpensive method that might also be used in countries with less access to expensive technology. In recent decades, non-coding RNA molecules (miRNAs) have emerged as a new class of regulatory genes involved in many cellular processes, such as development, differentiation, proliferation, apoptosis and the response to stress. In their mature form, these single-stranded small molecules are 19 to 25 nucleotides in length and are able to silence gene expression at the post-transcriptional level, thus inhibiting protein translation. They act by recognizing a sequence within the 3’ and/or 5’ untranslated region (UTR) of their mRNA target [8]. miRNAs can exert their action in cancers through both tumour suppression and oncogenic mechanisms [9,10]. Due to their structural characteristics, functions and tissue specificity, miRNAs have been proposed as a new class of biomarkers for the screening of different tumours. Recent discoveries have shown that miRNAs are also present in biological fluids, such as blood [11], urine [12], sputum [13] and saliva [14]; moreover, they were found to be aberrantly expressed in different human cancers and feature unprecedented levels of diagnostic specificity and sensitivity that are associated with tumour burden and malignant progression [15,16]. Additionally, circulating miRNAs are extremely stable against degradation by RNase, easily collected through non-invasive methods (blood sampling), resistant to repeated freeze/thaw cycles and easily identified by nucleic acid amplification techniques (real-time PCR). These factors also make their collection possible in third-world countries and underserved areas. Taken together, these findings highlight the potential usefulness of these molecules as promising biomarkers. To date, many studies have identified different circulating miRNAs as new biomarkers for BC detection; however, the results are discordant [17]. Moreover, they have not been assessed in conjunction with diagnostic imaging, which is used for quantifying the extent of the disease and provides information related to tumour aggressiveness representing an obligatory step for treatment planning. The use of diagnostic imaging to extract quantitative parameters related to the morphology, metabolism and functionality of tumours, as well as to correlate specific imaging parameters with tissue biomarkers, is an emerging research topic [18,19]. The aim of our study was to identify potential new disease-related circulating miRNAs with high diagnostic accuracy for BC and to elucidate the relationship between their circulating deregulation and tumour characteristics, including stage at presentation and the functional quantification of tumour metabolism and perfusion, by using same-day positron emission tomography/magnetic resonance (PET/MR) imaging. Since PET/MR scanners have been approved for use less than 8 years ago, with very few installed worldwide, PET/MR is currently clinically used for BC diagnosis and treatment planning in only an extremely limited number of highly specialized centres that have both the expertise of scanner use and a strong oncologic breast programme. In these centres, PET/MR is used for the whole-body and local staging of newly diagnosed BC cases that are amenable to curative resection, either without or after neoadjuvant treatments, and as problem-solving technology in complicated, more advanced cases. PET/MR is currently not mentioned in the National Comprehensive Cancer Network (NCCN) guidelines or in any other scientific organization/society guidelines despite its higher performances than those of any other non-invasive imaging modality. In fact, the accuracy of PET/MR staging outperformed that of PET/computed tomography (CT) (the most accurate of the routinely clinically approved imaging modalities) on a per patient analysis (98% versus 75%) and was also capable of demonstrating bony metastases not detected by same-day PET/CT in up to 12% of the cases [20,21]. These results have been confirmed by other authors who showed that, in a lesion-per-lesion analysis, PET/MR had higher sensitivity than PET/CT in detecting bony lesions (0.924 and 0.6923, respectively). Moreover, PET/MR also had a better sensitivity than PET/CT in detecting contralateral tumours (1 and 0.25, respectively) and all lesions together (0.89 and 0.77, respectively) [22]. To the best of our knowledge, this is the first study that aimed to correlate the deregulated expression of disease-related circulating miRNAs with the morphological, metabolic and functional parameters of BC lesions (tumour metabolism, cellular density and vascularisation) extracted through a hybrid PET/MR scanner. The plasma samples of BC patients and healthy donors were analysed for miRNA expression, and for each BC patient, PET/MR was used to ascertain the stage of the disease as well as the metabolic, diffusion and perfusion characteristics of the primary cancer. In silico studies and in situ hybridization (ISH) experiments were also performed to better understand miRNA expression in tissue and their cellular localization. Last, we correlated the expression levels of the validated miRNAs with immunohistochemical markers of BC, stage of disease and PET/MR-derived functional and metabolic biomarkers of primary cancer.

## 2. Results

### 2.1. Identification and Validation of a Plasma miRNA Signature for Breast Cancer Detection

**Figure 1:**
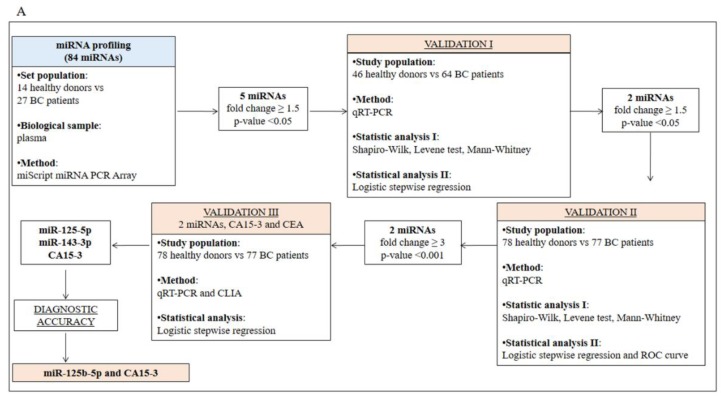

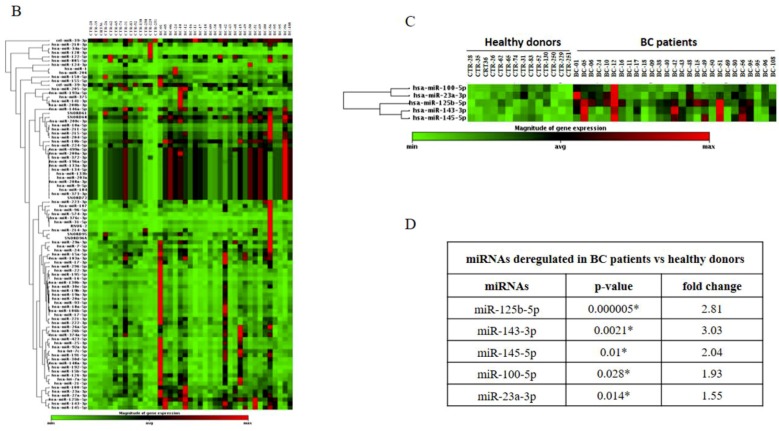
Study design for the identification of circulating miRNAs and protein biomarkers able to discriminate breast cancer (BC) patients from healthy donors (**A**). miRNA expression analysis in a set population of 14 healthy donors (CTR) and 27 BC subjects. Expression analysis of 84 miRNAs by using a 96-well miScript miRNA PCR Array (**B**). * miRNAs significantly upregulated (*p* < 0.05) in BC patients vs. healthy donors (**C**,**D**).

**Figure 2:**
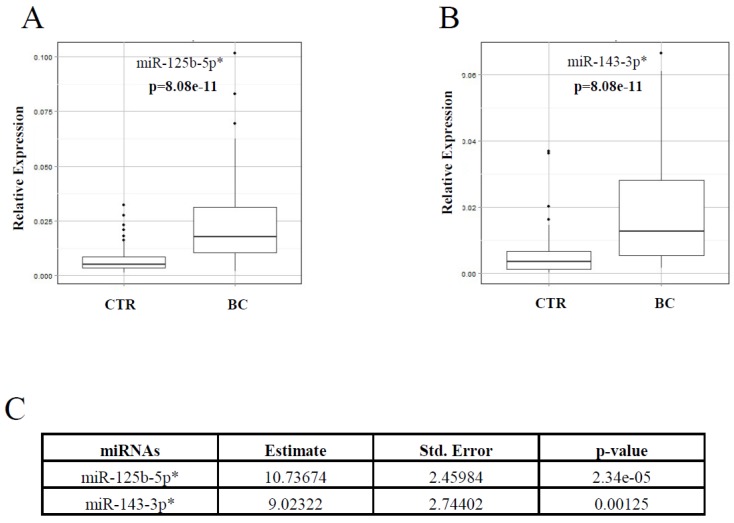
Validation II. Relative expression of miRNAs selected in a study population of 78 healthy donors (CTR) and 77 breast cancer (BC) subjects. Box plot analyses performed to show the relative expression of miR-125b-5p (**A**) and miR-143-3p (**B**) in the plasma samples of healthy donors vs. BC patients. Logistic stepwise regression analysis (**C**). * miRNAs that significantly (*p* < 0.05) estimate the probability of having BC.

The study design is illustrated in Figure 1A. The healthy and BC subjects were age-matched (*p*-value = 0.157), and the clinicopathological characteristics of the study participants are reported in Table 1. By using the miScript miRNA PCR Array, we found that five (miR-125b-5p, miR-143-3p, miR-145-5p, miR-100-5p and miR-23a-3p; Figure 1B–D) out of the 84 miRNAs analysed were significantly (*p*-value < 0.05) upregulated in the plasma samples of 27 BC patients vs. 14 healthy donors, with a fold change ≥1.5. RT-PCR was conducted to confirm the array results in a study population of 46 healthy donors and 64 naïve BC patients (Supplementary Figure S1). Then, a logistic regression model (Supplementary Table S1A) identified miR-125b-5p and miR-143-3p as the circulating miRNAs able to effectively discriminate affected from unaffected subjects. A logistic stepwise regression model confirmed these results (Supplementary Table S1B). Moreover, the results were corroborated by expanding the case studies to 78 healthy donors and 77 naïve BC patients (Figure 1A, Validation II). In fact, as shown in Figure 2A,B, we confirmed that miR-125b-5p and miR-143-3p were significantly upregulated in the plasma of BC patients with a fold change of 3.3 and 3.2, respectively. These results were confirmed by logistic stepwise regression analysis (Figure 2C).

### 2.2. Diagnostic miRNA Signature-Based Model Compared with Established BC Markers

**Figure 3:**
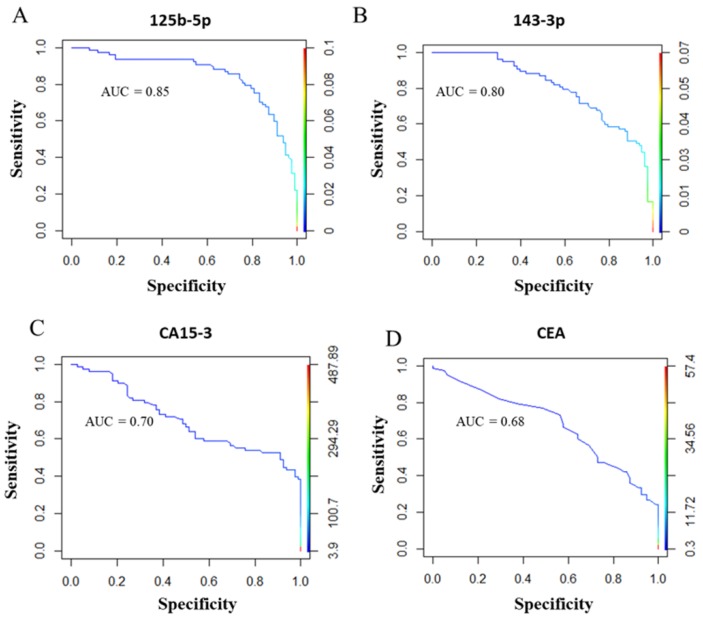
ROC curve analyses and AUC values of miR-125-5p (**A**), miR-143-3p (**B**), CA15-3 (**C**), and CEA (**D**) for discriminating BC patients from healthy controls in 76 BC patients and 78 healthy donors. ROC: Receiver operating characteristic; AUC: area under the ROC curve; CEA: carcinoembryonic antigen.

Receiver operating characteristic (ROC) curve analyses showed that miR-125b-5p (area under the ROC curve (AUC) = 0.85) and miR-143-3p (AUC = 0.80) were able to discriminate BC patients from healthy donors (Figure 3A,B). Then, we compared the AUC value of the selected miRNAs with the AUC value of the traditional BC biomarkers CA15-3 and CEA. As reported in Figure 3C,D, the AUC values of CA15-3 (0.70) and CEA (0.68) were lower than those calculated for miR-125b-5p and miR-143-3p. Based on these results, a logistic stepwise regression analysis was performed considering the miR-125b-5p, miR-143-3p, CA15-3 and CEA molecules as independent variables (Figure 1A, Validation III). According to Table 2, miR-125b-5p, miR-143-3p and CA15-3 were significantly correlated with the disease, although the statistical significance of CA15-3 was lower than that of the miRNAs. Based on these findings, the diagnostic accuracy of each biomarker was evaluated. To this end, the cut-off values of miR-125b-5p and miR-143-3p were calculated, and the clinical cut-off values of CA15-3 and CEA were already known (CA15-3 = 35 UI/mL and CEA = 3 UI/mL). Using the “closest to top-left” method, we defined the cut-off value of miR-125b-5p as 0.0090 and that of miR-143-3p as 0.0064, both related to relative expression. Among the analysed molecules, miR-125b-5p showed the highest diagnostic accuracy (Table 3).

### 2.3. Co-Expression Analysis of Circulating miR-125b-5p, miR-143-3p and miR-145-5p and Their Association with Immunohistochemical Markers of BC 

**Figure 4:**
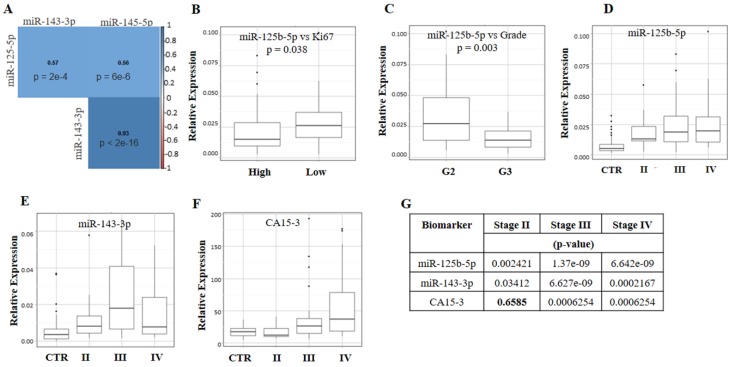
Correlation studies. Co-expression analyses of miR-125b-5p, miR-143-3p and miR-145-5p (**A**). Expression levels of miR-125b-5p vs. Ki67 (low <20%; high ≥20%) (**B**), and grade (**C**). Expression levels of miR-125b-5p (**D**), miR-143-3p (**E**), and CA15-3 (**F**) vs. stage. Statistical significance (**G**). Non-significant results are reported in bold.

Although miR-145-5p was not able to estimate the probability of pathology (Supplementary Table S1), it is transcribed from a putative miR-143/145 cluster on chromosome 5 in humans (5q33). Therefore, co-expression analyses by Spearman’s test were performed not only for miR-125b-5p and miR-143-3p but also for miR-145-5p. As reported in Figure 4A, we found that miR-143-3p and miR-145-5p were strongly co-expressed (*ρ*: 0.93; *p*-value < 2 × 10^−16^) in the plasma samples of BC patients, and the expression levels of miR-125b-5p were significantly correlated with those of miR-143-3p (*ρ*: 0.57; *p*-value = 2 × 10^−4^) and miR-145-5p (*ρ*: 0.56; *p*-value = 6 × 10^−6^), although to a limited extent.

The immunohistochemical (IHC) reports of the enrolled BC patients were used to determine the receptor status (oestrogen, progesterone, and human epidermal growth factor receptor 2), cellular differentiation status (grade) and proliferation index (Ki67) of the tumour lesions. No association was found between the expression levels of miR-125-5p, miR-143-3p and miR-145-5p and the hormonal receptor status of the lesions (Estrogen Receptor positive or negative: ER+/−, Progesteron receptor positive or negative: PR +/− and Human epidermal growth factor receptor positive or negative: HER2 +/−; Supplementary Table S2) or with tumour subtypes (all *p*-values > 0.05). Nevertheless, only miR-125b-5p was significantly associated with the Ki67 proliferation index (Figure 4B, *p*-value = 0.038) and the differentiation status of tumour cells (Figure 4C, *p*-value = 0.003). Specifically, we found that in the presence of a low Ki67 index and a low grade, the expression levels of miR-125b-5p were increased, suggesting its inverse correlation with the aggressiveness of the disease.

### 2.4. Correlation Analysis of Circulating miR-125b-5p, miR-143-3p and miR-145-5p with Quantitative Imaging Parameters of Tumour Lesions

The BC patients enrolled in this study underwent same-day contrast-enhanced fluorodeoxyglucose (CE-FDG)-PET/MR and blood collection to better correlate circulating miR-125-5p and miR-143-3p with the morphological, metabolic and functional imaging features of BC (Figure 4). No significant correlations were found between the miRNA expression levels and lesion size (Supplementary Table S2). The plasma levels of miR-125b-5p increased significantly at stage II, reaching higher expression levels at stage III and remained unchanged at stage IV compared to stage III (Figure 4D,G). This trend confirmed that the expression levels of miR-125b-5p were variable and depended on the severity of the disease. Additionally, the expression levels of miR-143-3p (Figure 4E,G) became significantly higher in stage III and drastically decreased in stage IV, reaching expression values close to those of healthy donors. This result suggests that at stage IV, this molecule is not required for the maintenance of the pathology. It is known that CA15-3 levels increase in the late stage of the disease, so its relationship with clinical staging was also evaluated. As reported in Figure 4F,G, in our cohort, CA15-3 was not able to diagnose stage II, but its levels increased significantly at stage III, reaching the maximum concentration at stage IV. This was in agreement with the known performance of CA15-3. In addition, no correlations were found between CA15-3 and grade (*p*-value = 0.761) or subtypes (*p*-value = 0.450).

Correlation analyses between miR-125b-5p and miR-143-3p levels and PET/MR-based tumour biomarkers were performed by grouping the data according to staging. Only the statistically significant results were reported (Table 4); we found a strong and significant correlation between circulating miR-143-3p and the mean initial area under the concentration curve (iAUC_mean_) and the mean reverse efflux volume transfer constant (Kep_mean_) (for both, ρ: 0.943, *p*-value = 0.005) in the stage II group. Perfusion parameters such as iAUC and Kep are generally correlated with tumour vascularisation [23,24], suggesting that the plasmatic overexpression of miR-143-3p in BC patients might correlate with angiogenesis. The correlation between iAUC and miR-143-3p persisted at stage III but was lost at stage IV, where its relative expression drastically decreased (see Figure 4B). Notably, there was a strong correlation index between miR-143-3p and the maximum standardized uptake value (SUV_max_) (ρ: 0.829 and *p*-value = 0.042) at stage II, although the statistical significance was borderline. SUV is a biomarker of tumour metabolism. This result together with those reported above suggest that miR-143-3p could be correlated with tumour aggressiveness. miR-125-5p was significantly inversely correlated with the mean forward volume transfer constant (Ktrans_mean_) and the proliferation index Ki67 at stage IV (Table 4). Ktrans_mean_, like iAUC and Kep, is a perfusion parameter linked to tumour vascularisation [24]. Since the highest plasma concentration of miR-125b-5p was associated with a low Ki67 index (Figure 4B) and grade G2 (Figure 4C) and inversely correlated with Ktrans_mean_ (Table 4), these results suggest that the highest plasma values of miR-125b-5p might predict a better prognosis.

### 2.5. In Silico Studies

Large publicly funded projects have generated extensive and freely available multi-assay data resources. To date, The Cancer Genome Atlas (TCGA) represents the largest available resource for multi-assay cancer genomics data that includes ~30000 cases of major primary cancers, including BC (TCGA-BRCA) [25]. To evaluate whether the upregulation of circulating miR-125b-5p, miR-143-3p and miR-145-5p in the plasma samples of BC patients reflected their tumour tissue expression, we performed in silico studies by using the TCGA-BRCA database. We also included miR-145-5p in order to perform co-expression analyses. We found that in BC tissue specimens, the expression of miR-125b-5p, miR-143-3p and miR-145-5p was significantly lower compared to that in normal tissues (Supplementary Figure S2A) and that their downregulation was not correlated with the stage of the disease (Supplementary Figure S2B). 

In addition, we found that the downregulation of miR-143-3p and miR-145-5p in BC tissues (Supplementary Figure S2C) showed a lower correlation index compared to that we found in the plasma samples of BC patients (compare Figure 4A with Supplementary Figure S2C: *ρ*: 0.93 vs. *ρ*: 0.28). Taken together, these results showed an opposite expression trend of miR-125-5p, miR-143-3p and miR-145-5p in cancer tissue versus plasma.

**Figure 5:**
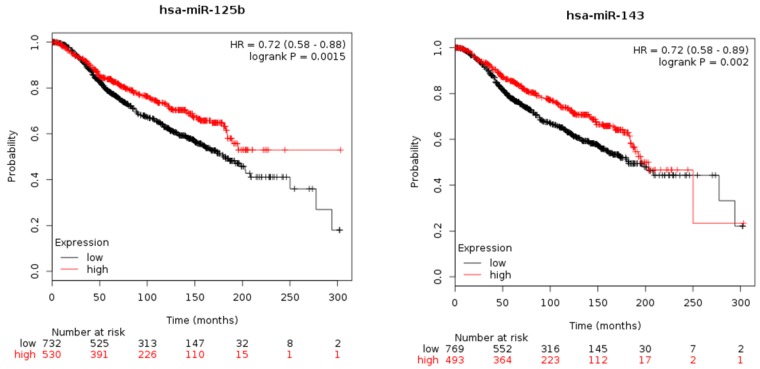
Kaplan-Meier plots, which are based on breast cancer data from the METABRIC database, illustrate the survival probability for patients with low or high miR-125b (upper panel) and miR-143 (lower panel) expression levels in breast cancer. Hazard ratios (HRs) and *p*-values (log-rank test) were generated.

Since we were unable to monitor patients during follow-up, a survival analysis was performed for both miR-125b-5p and miR-143-3p by using the miRpower software (http://kmplot.com/analysis/index.php?p=service&cancer=breast_mirna). This software uses publicly available BC data from different databases, including data from large databases such as TCGA and Molecular Taxonomy of Breast Cancer International Consortium (METABRIC). To generate Kaplan-Meier curves, we selected the option to auto select the best cut-off for defining high and low groups. The algorithm computes all possible cut-off values between the lower and upper quartiles and uses the best performing threshold. The hazard ratios (HRs) and *p*-values (log-rank test) were calculated. By using the TCGA dataset, the Kaplan-Meier plots were not statistically significant for miR-125b-5p or miR-143-3p (data not shown), so the METABRIC dataset was used. As reported in Figure 5, we found that high tissue expression levels of miR-125b-5p were associated with the best prognosis (upper panel), and this result was in agreement with those obtained correlating this miRNA with grade, Ki67 and Ktrans_mean_. The effect of high expression levels of miR-143 on the survival of BC patients varied with age, and higher values contributed to worse prognoses starting at 250 months (lower panel). This result was in part in agreement with those obtained correlating this miRNA with the imaging parameters linked with tumour aggressiveness. It is necessary to point out that the miRpower software did not discriminate miR-143-3p from miR-143-5p, grouping them in miR-143, and miR-125b-5p from miR-125b-3p, grouping them in miR-125b.

### 2.6. Cellular Origin of miR-125b-5p, miR-143-3p and miR-145-5p in Normal and BC Tissues

**Figure 6:**
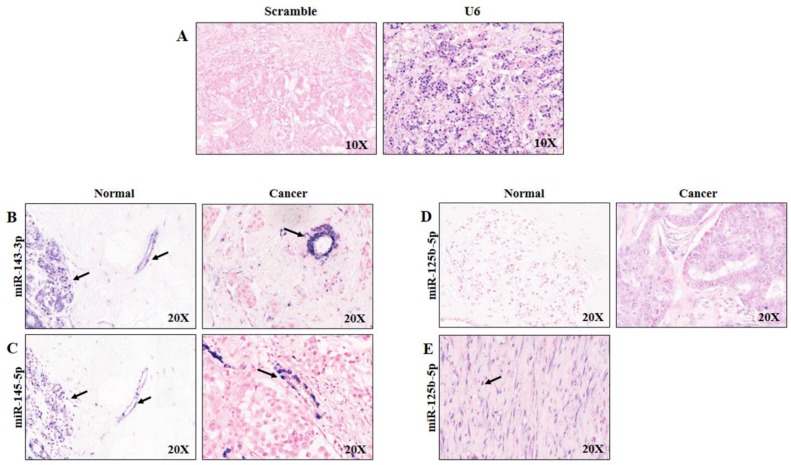
In situ hybridization staining patterns in benign and malignant breast tissues. Scrambled miR negative control probe and U6 positive control probe (**A**). miR-143-3p in normal (left) and BC tissues (right) (**B**). miR-145-5p in normal (left) and BC tissues (right) (**C**). miR-125b-5p in normal (left) and BC tissues (right) (**D**). miR-125b-5p in stromal tumour tissue (**E**). BC: breast cancer.

To corroborate the obtained results and confirm the in silico data, we performed ISH experiments. We hypothesized that release of miR-125b-5p, miR-143-3p and miR-145-5p into the bloodstream did not originate from tumour cells. As reported in Figure 6, the results confirmed that the expression of miR-143-3p (B) and miR-145-5p (C) was low or absent in epithelial tumour tissue (right) but high in endothelial cells, highlighting their vascular origin, as anticipated by correlation studies among miRNAs with image biomarkers. In addition, in benign breast tissue, miR-143-3p stained in glandular epithelial and endothelial cells (Figure 6B, left), and miR-145-5p was expressed with the strongest staining intensity in myoepithelial cells and endothelial cells (Figure 6C, left panel). Moreover, miR-125b-5p was weakly expressed in normal tissue (Figure 6D, left) and low-to-absent in neoplastic cells (Figure 6D, right). This supported the hypothesis that miR-125b-5p could have a stromal origin. This hypothesis was further confirmed by the result shown in Figure 6E, in which miR-125b-5p expression occurred in cancer-associated fibroblasts. Taken together, these results confirmed that the BC plasma upregulation of miR-125-5p and miR-143-3p could be related to their overexpression in endothelial and fibroblast cells and not in tumour epithelial cells.

### 2.7. Combining Diagnostic test Results to Increase Accuracy

**Figure 7:**
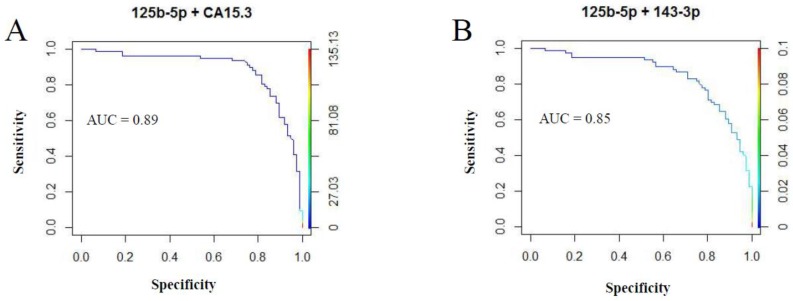
ROC curve analyses and AUC values combining the profiles of miR-125b-5p and CA15-3 (**A**) and miR-125-5p and miR-143-3p (**B**) for discriminating BC patients from healthy patients. ROC: Receiver operating characteristic; AUC: area under the ROC curve; BC: breast cancer.

Since the relationship between upregulated circulating miRNA expression and the functional characteristics of tumour lesions was clarified, we next sought to optimize their diagnostic role by evaluating a combined detection of miR-125b-5p and miR-143-3p with CA15-3. As reported in Figure 7A, we found that combining the diagnostic test results of miR-125b-5p and CA15-3 improved the accuracy (AUC = 0.89) compared to the same molecules analysed individually (see Figure 3: miR-125-5p, AUC = 0.85; CA15-3, AUC = 0.68). In contrast, combining miR-143-3p and CA15-3 reduced the diagnostic accuracy (data not shown). Additionally, the combined analysis of miR-125b-5p and miR-143-3p did not improve the accuracy (Figure 7B, AUC = 0.85) in comparison to the same molecules analysed individually (see Figure 3: miR-125-5p, AUC = 0.85; miR-143-3p, AUC = 0.80).

## 3. Discussion

The detection of BC is pivotal for proper patient management, especially at an early stage. However, the currently utilized technology, which is heavily based on screening mammography and ultrasound (in specific patient populations), is imperfect, with limited accessibility in underdeveloped/underserved areas and might be economically challenging. Therefore, there is a compelling need to identify new biomarkers that might be diagnostically advantageous. Due to their structural and functional characteristics, microRNAs have been proposed as a new class of biomarkers for cancer screening; in fact, they are stable, deregulated, and found in different biological fluids, including blood that can be easily withdrawn in the field and subsequently processed in centralized laboratories. To date, circulating biomarkers that are able to diagnose BC with high specificity and sensitivity are lacking, so our study aimed to (i) identify new circulating miRNAs for the diagnosis of BC; (ii) to elucidate the relationship between their circulating deregulation and the functional characteristics of tumour lesions for in vivo investigation through PET/MR; and (iii) understand the link between circulating miRNA deregulation and their origin in the primary tumour.

By using the miScript miRNA PCR Array, we found that five miRNAs (miR-125b-5p, miR-143-3p, miR-145-5p, miR-100-5p and miR-23a-3p) were significantly (*p* ≤ 0.0005) overexpressed in the plasma samples of BC patients compared to healthy subjects and defined miR-125b-5p and miR-143-3p as the best molecules able to estimate the probability of having the disease. The circulating levels of miR-125b-5p and miR-143-3p are of intense debate in the scientific literature, especially in BC. Matamala et al. [26] found that circulating miR-143 was upregulated in BC patients; on the contrary, some studies showed a downregulation of circulating miR-143 in BC patients [27,28,29,30], and others found no significant deregulation of circulating miR-125b and miR-143 in BC patients [30]. These discrepancies could be explained by the usage of serum rather than plasma [31]. Regardless of whether the choice of the biological sample can affect the result, different studies reported the upregulation of miR-125b in the serum samples of BC patients [32,33,34]. These discrepancies highlight the need to standardize protocols, including sample collection, storage, choice of the starting biological material, and processing. To better understand the biological basis and clinical implications of miR-125b-5p and miR-143-3p, we performed a correlation analysis between the circulating expression of the deregulated disease-related miRNAs and the metabolic and functional characteristics of the primary tumour, also taking into consideration the TNM (Tumour Lymph Nodes Metastasis) classification of malignant tumours stage of the disease. For this purpose, we employed biomarkers extracted from a hybrid clinically approved PET/MR scanner. We decided to use PET/MR since it couples two modalities (FDG-PET and MR) capable of sampling different biological features of cancers in vivo, specifically glucose metabolism through PET and cellular density plus tissue perfusion through MR. The advantages arising from the simultaneous acquisition of PET- and MR-derived biomarkers in investigating BC biology have been shown by some exploratory studies that demonstrated the capability of PET/MR to predict BC subtypes, including those with a better prognosis [19,35]. In this study, we found that the expression of circulating miR-143-3p was strongly and significantly correlated with iAUC_mean_ at stage II (ρ: 0.943, *p*-value = 0.005) and at stage III (ρ: 0.938, *p*-value = 0.044) and with Kep_mean_ at stage II (ρ: 0.943, *p*-value = 0.005). Both iAUC_mean_ and Kep_mean_ are perfusion parameters that provide information on tumour vascularisation, suggesting that miR-143-3p overexpression in the plasma samples of BC patients could be linked to tumour angiogenesis. Our hypothesis was in agreement with the editorial comment of Almeida and Calin [36] that argued the possible critical role of these miRNAs in the neoangiogenesis of lung cancer. To confirm this hypothesis and to further validate our results, we performed ISH experiments on BC and normal breast tissue specimens and found that miR-143-3p had a clear vascular endothelial origin. In addition, we also found that miR-143-3p was correlated with SUV_max_ at stage II (ρ: 0.829, *p*-value = 0.042). The SUV parameter reflects the metabolic activity of tumours, so we speculate that miR-143-3p could have a role in neoangiogenesis and that its overexpression in the plasma samples of BC patients could be correlated with the aggressiveness of the disease, both in terms of tumour vascularisation and metabolic activity. This hypothesis was in part corroborated by the survival analysis performed *in silico* by using the miRpower software. 

Another interesting result correlated the expression levels of miR-125b-5p with IHC biomarkers and imaging parameters. We concluded that miR-125b-5p was inversely associated with the proliferation index Ki67 (*p*-value = 0.038), grading (*p*-value = 0.003) and the perfusion imaging parameter Ktrans_mean_ (ρ: −0.421, *p*-value = 0.040) and that its expression occurred in cancer-associated fibroblasts. These findings suggest that the highest concentration of miR-125b-5p could be correlated with a better prognosis, and the survival analysis confirmed this hypothesis. This is in agreement with the “seed and soil” hypothesis, which postulates that different components of the tumour microenvironment (the soil) may have stimulatory or inhibitory effects on tumour progression (the seed) [37], and with recent studies showing that miR-125b-5p overexpression in BC cell lines could inhibit cell proliferation, migration and invasion [38,39].

One of the limitations of this study is the sample size (*n* = 77 BC patients). In fact, the heterogeneity and complexity of BC make it challenging to find a unique circulating signature of pathology with high diagnostic accuracy. Each circulating biomarker released in the blood of affected patients exists as a consequence of genetic and/or epigenetic alterations linked to the pathology. Breast carcinoma is divided into different subtypes with different genetic characteristics [40,41], which can affect the release of specific miRNAs in the blood. Moreover, the variety of biomarkers and the difference in concentration in the blood might be influenced by the stage, so the analysis of a cohort not homogeneous for staging could be a limitation, and the BC patients included in this cohort are lacking stage I and grade 1 (one patient for each condition). Therefore, a limitation of our study is the paucity of stage I and II cancers. However, this is unavoidable in studies that recruit clinically justified PET acquisition since, as per the Clinical Practice Guidelines in Oncology (NCCN guidelines), PET is not indicated in the initial work-up of clinical stage I, II, or operable stage III (NCCN Guidelines Version 1.2019, accessed on 22th May 2019).

This staging group heterogeneity might lead to different results in different cohorts. Another limitation was the inability of monitoring patients during follow-up to better understand the prognostic and predictive role of miR-125b-5p and miR-143-3p. Nevertheless, the strengths of this study included the analysis performed on naïve patients as well as the availability of the same-day imaging parameters that provided information related to the morpho-functional characteristics of the primary cancers to correlate circulating miRNAs with in vivo quantified tumour biology. We performed a combined detection of miR-125b-5p and miR-143-3p with CA15-3 to improve the diagnostic accuracy. As suggested by guidelines, CA15-3 is not recommended for BC diagnosis [42,43], and our result (CA15-3_AUC_ = 0.70) was in agreement with those of Liu and colleagues (CA15-3_AUC_ = 0.71 [44]), Zaleski and colleagues (CA15-3_AUC_ = 0.72 [45]) and Zajkowska and colleagues (CA15-3_AUC_ = 0.70 [46]). Nevertheless, the coupled analysis of miR-125b-5p and CA15-3 increased the diagnostic accuracy up to AUC = 0.89. To the best of our knowledge, this is one of the best results published so far and the first study that combines the expression of a circulating miRNA with the expression of an established BC tumour marker. The combined detection of plasma miR-125b-5p and serum CA15-3 improved the diagnostic accuracy of BC detection up to ~90%, suggesting that this signature could be used for the diagnosis of BC. In addition, for stage II, the expression levels of CA15-3 were not correlated with the pathology (*p*-value = 0.658), whereas those of miR-125b-5p were (*p*-value = 0.002). The combined analysis of miR-125b-5p and CA 15-3 not only improved diagnostic accuracy in its entirety but also provided additional diagnostic information at less advanced pathological stages, suggesting the use of miRNAs as potential diagnostic circulating biomarkers that warrants consideration by further validation studies.

## 4. Materials and Methods

### 4.1. Participants and Study Design

This Health Insurance Portability and Accountability Act–compliant prospective study was approved by the institutional review board. All procedures performed in studies involving human participants were in accordance with the ethical standards of the institutional and/or national research committee and with the principles of the 1964 Declaration of Helsinki and its later amendments or comparable ethical standards. Informed consent was obtained from all individual participants included in the study. For study enrolment, all participants provided written informed consent before undergoing imaging PET/MR and blood collection. Between September 2012 and November 2015 at the IRCCS SDN Institute, 221 female Caucasian patients with BC who underwent a clinically indicated same-day CE-FDG-PET/MR and blood collection were evaluated for inclusion in this study. Plasma haemolysation was evaluated as described by Kirschner MB and colleagues [47]. The inclusion criteria for BC patients were as follows: (i) diagnosis of BC by core biopsy report; (ii) absence of any prior surgical or pharmacological treatment for BC (naïve); (iii) negative previous personal oncological history; (iv) age > 18 years; (v) CE-FDG-PET/MR and blood collection were performed on the same day; and (vi) fasting for at least 8 hours. The exclusion criteria for BC patients were as follows: (i) pregnancy; (ii) blood glucose levels > 140 mg/dL (7.77 mmol/L); (iii) artefacts affecting PET/MR images; (iv) standard contraindications for MR; (v) performance of CE-FDG-PET/MR and blood collection on different days; and (vi) plasma haemolysation. Blood samples of age-matched healthy donors were collected; their inclusion criteria were as follows: (i) >18 years of age; (ii) negative previous personal oncological history; (iii) absence of suspected BC as confirmed by ultrasound and/or mammography during the last 6 months from enrolment; iv) absence of a family history of BC; and (v) fasting for at least 8 hours. The exclusion criteria for healthy donors were the presence of one or more of the criteria reported above, including haemolysed plasma. We recruited 77 BC patients and 78 healthy donors for a total of 155 plasma/serum samples. The clinicopathological characteristics of the enrolled patients are reported in Table 1, and the whole study design is shown in Figure 1A.

This study was approved by the institutional Ethics Committee (Protocol Number: Prot2-11, approved 06/07/2011 by Ethical Committee IRCCS Fondazione SDN). This article does not describe any studies with animals performed by any of the authors

### 4.2. PET/MR Data Acquisition

PET/MR was performed with a Biograph mMR imager (Siemens Healthiness, Erlangen, Germany). First, a whole-body protocol was used to assess the overall extent of cancer and provide the NM stage. Thereafter, a dedicated breast protocol to provide both metabolic and functional biomarkers of primary cancer and its T stage was acquired. For total body acquisition, we used a 16-channel head-neck coil and three or four 12-channel body coils depending on the patient’s height; for breast acquisition, a 4-channel breast coil was employed. Acquisitions were performed according to previously described protocols [19,20,35,48,49]. All patients fasted for at least 8 h before the procedure. They received 401 ± 32 MBq (mean ± standard deviation) of 18F-FDG intravenously. After a 60-min incubation time, PET and MR data were acquired simultaneously. The mean total time for the PET/MR examination was 107.87 ± 18.92 min [19].

### 4.3. Data Processing and Multiparametric Analysis

The PET data obtained from the PET/MR examinations were processed with comparable reconstruction and correction algorithms. Emission data were corrected for randomness, dead time, scatter, and attenuation.

Lymph node involvement and distant metastases were assessed on the entire dataset of PET/MR sequences for the purpose of NM staging. The primary tumour size and infiltration of neighbouring structures were evaluated on the dedicated breast PET/MR protocol for T staging [50].

The dynamic contrast-enhanced (DCE)-MRI images were processed through commercially available and clinically approved software for estimating tissue perfusion (Tissue 4D, Siemens Healthiness) according to the same established procedures provided by the manufacturer manual and widely used in the published literature. The pharmacokinetic modelling is based on a two-compartment Toft’s model that allows for the calculation of the following: the transfer constant between vascular, extravascular, and extracellular space (EES) (Ktrans); volume of EES (Ve); constant reflux between EES and blood plasma (kep); initial area under the concentration curve (iAUC) [51,52]. Ktrans is a parameter related to vessel permeability and tissue blood flow. The volume of the extravascular extracellular space Ve is a marker of cell density, kep is a transfer constant from the extracellular/extravascular space to plasma, and iAUC is related to the blood volume in the tissue of interest [51].

After automatic motion correction and registration of the pre- and post-contrast acquisitions, T1 mapping was automatically obtained from the multiple flip angle pre-contrast part of the DCE-MRI sequence. Arterial input function (AIF) was related to the gadolinium dose injected and automatically modelled by a bi-exponential function using an intermediate population-derived mode provided by the software. On each single slice, a region-of-interest (ROI) was manually plotted around the tumour excluding the neighbouring vessels (internal mammary arteries and heart chambers) to compute the MR perfusion biomarker maps that were stored in the system.

For the subsequent tumour ROI analysis, PET/MR datasets (PET acquisition, dynamic axial T1 weighted post-contrast, dynamic axial T1 weighted subtracted contrast-enhanced images, axial T2 weighted sequences, axial apparent diffusion coefficient (ADC) map, and perfusion maps for Ktrans, Ve, kep and iAUC) were simultaneously evaluated on a clinically approved hybrid imaging workstation (Syngo.via, Siemens Healthiness) allowing the visual and quantitative comparison of the multiparametric data.

The maximum standardized uptake value (SUVmax) was automatically calculated by using an iso-contour automatic volume-of-interest (VOI) method of tissue delineation that selected a fixed threshold fraction (40%) of the peak activity in the tumour.

To obtain Ktrans, Ve, Kep and iAUC, the subtracted dynamic post-contrast T1-weighted series that best visualized the tumour was chosen. ROIs were manually drawn around the tumour on each image of that series, copied and then pasted on the corresponding Ktrans, Ve, Kep and iAUC maps. The values of all the ROIs were averaged. To obtain the ADC values, the ROIs were drawn on each image of the high-b value (800 s/mm²) diffusion-weighted imaging (DWI), copied and then pasted on the ADC maps. The values of all the ROIs were averaged.

While the ROIs were drawn around the tumours, care was taken to avoid including large feeding vessels and areas of frank necrosis, as seen on T2 weighted and subtracted dynamic post-contrast T1-weighted images.

The maximal diameter of the tumour was measured on every single slice of the reference subtracted dynamic post-contrast T1-weighted images. The measurements were performed by a radiologist and a nuclear medicine physician with more than 5 years of experience. The whole-body and breast PET/MR protocols were derived from the published literature [20,35].

### 4.4. Plasma Sampling, RNA Extraction and Reverse Transcription

Blood samples were collected immediately before FDG injection. For each patient, a total of 10.5 mL of venous blood was collected in BD Vacutainer^®^ 3 mL ethylenediaminetetraacetic acid (EDTA) tubes (Becton Dikinson, Franklin Lakes, NJ, USA) and 7.5 mL serum separating tubes. Blood was processed within 1 h of harvest. Plasma and serum were obtained from the whole blood samples by centrifugation at 1900× *g* for 10 min at 4 °C. The supernatant was further centrifuged at 16,000× *g* for 10 min at 4 °C and stored in aliquots of 0.5 mL at −80 °C until analysis. The samples were stored at −80 °C at the SDN Biobank (IRCCS DSN, Naples, Italy) [53]. The extraction of total RNA from 200 µL of plasma was performed within 1 year of storage at −80 °C using an miRNeasy Serum/Plasma Kit (Qiagen, Hilden, Germany) according to the manufacturer’s instructions. Briefly, 3.5 µL of *Caenorhabditis elegans* miR-39 (cel-miR-39, 1.6 × 108 copies/µL) was added as the spike-in control after the denaturing solution. Total RNA (including miRNAs) was eluted in 14 µL of RNase-free water. Reverse transcription was performed using the miScript II RT Kit (Qiagen, Hilden, Germany) according to the manufacturer’s instructions. Because extracellular miRNAs are less than 1% of the total RNA recovered [54] their concentration is often under the detection limits of spectrophotometric devices, so we decided to use a fixed volume rather than a fixed miRNA amount for qRT-PCR. To this aim, 1.5 µL of total RNA was added to the HiSpect Buffer (Qiagen, Hilden, Germany), nucleic acid mix and reverse transcriptase in a final volume of 20 µL. Reactions were incubated for 60 min at 37 °C and then for 5 min at 95 °C to inactivate the miScript Reverse Transcriptase mix (Qiagen, Hilden, Germany). At the end of the reactions, the resulting cDNAs were stored undiluted at −20 °C. The cycling conditions for real-time PCR were as follows: 15 min at 95 °C as the initial activation step, followed by 3-step cycling including denaturation (15 s at 94 °C), annealing (30 s at 55 °C) and extension (30 s at 70 °C) for a total of 40 cycles.

### 4.5. miRNA Microarray and Validation by Real-Time PCR (qRT-PCR)

By using the miScript miRNA PCR Array (Qiagen; MIHS-106Z), the expression profile of 84 human miRNAs (Supplementary Table S3) from the plasma samples of naïve BC patients and healthy donors were screened according to the manufacturer’s protocol. The miScript miRNA PCR Array profiles the expression of 84 human miRNAs known to be deregulated in different cancers and are best characterized in miRBase. miRNA profiles were initially checked against a set population of 41 plasma samples (14 plasma samples from healthy donors and 27 from BC patients at diagnosis) on a MyiQ PCR system (Bio-Rad, Hercules, CA, USA). To identify interindividual variability, we did not pool plasma samples from each group (BC patients and healthy donors), but each sample was individually analysed by miRNA PCR arrays. The 96-well array also included *n* = 2 miRNA isolation controls, *n* = 6 “Housekeeping” snRNAs, *n* = 2 miRNA Reverse Transcription Controls (miRTC) and *n* = 2 Positive PCR Controls (PPC). Briefly, 200 µL of RNase-free water was added to each 20 µL of reverse transcription reaction, and 100 µL of diluted cDNA was used to prepare a reaction mix according to the manufacturer’s protocol. For data analysis, the Baseline and Threshold settings, which were the same across all PCR runs, were from cycle 2 to cycle 15 for Baseline and 20 for Threshold. By using a data analysis tool (GeneGlobe Data Analysis Center, Qiagen, Hilden, Germany), the threshold cycle (CT) values were exported, and the steps performed by the software were as follows: any CT value ≥ 33 was considered negative; if the RNA sample was of high quality, the cycling programme was correctly run and the threshold was correctly defined, the value of the positive PCR control wells from the CT samples (CTPPC) should be 19 ± 2 or 15 ± 2; if the CT values of the reverse transcription control are less than 7, no inhibition of the reverse transcription reaction is apparent; the ΔCT value for each mature miRNA profiled is calculated using the formula ΔCT = CTmiRNA - CTnormalizer. The relative expression for each miRNA was calculated as 2^−ΔCT^, and the criteria for the selection of miRNAs differentially expressed between the two populations with a statistical significance were a fold change ≥1.5 and a *p*-value < 0.05. Data validations were performed by qRT-PCR screening for only the following molecules: miR-125b-5p, miR-143-3p, miR-145-5p, miR-100-5p and miR-23a-3p. The Qiagen miScript SYBR Green PCR Kit was used for qPCR according to the manufacturer’s protocol on the Bio-Rad MyiQ PCR system. To determine the best standard genes to normalize our data, BestKeeper software was used [55]. By analysing *n* = 41 plasma samples, we found that the best normalizer included in the miScript miRNA PCR Array was the exogenous cel-mir 39. The MiScript Primers Assay was purchased from Qiagen (Qiagen, Hilden, Germany).

### 4.6. TCGA Data Processing

TCGA Breast Invasive Carcinoma (TCGA-BRCA project, February 2018) transcriptome profiling data (miRNA Expression Quantification) and clinical metadata were extracted from the Genomic Data Commons (GDC) portal (https://portal.gdc.cancer.gov/) for primary tumour (*n* = 1096 files for 1078 BC cases) and normal solid tissue (*n* = 104 files for 104 normal cases) using the open source R software and the TCGAbiolinks R package [56]. For the miRNA datasets, we selected the level-3 miRNA-Seq data, which were produced on Illumina HiSeq 2000 sequencers (Illumina Ventures, San Diego, CA, USA) The miRNA-Seq expression level-3 data contains raw read counts (*n* = 1881 miRNAs). The miRNA raw counts were filtered as follows: we calculated the mean value for duplicated BC cases and filtered out miRNA counts with null expression values on both conditions (tumour/normal) for a total of 1625 filtered counts. We considered only 1625 filtered miRNAs for the normalization step. Filtered raw counts were normalized using the Upper Quartile approach, and the patients with “−Inf” normalized values were removed for a total of 1076 tumours. The normalized counts were used to evaluate miR-125b-5p, miR-143-3p and miR-145-5p expression levels (tumour vs. normal) as log2-transformed relative expression values. We performed a Wilcoxon test to assess the statistical significance between tumour and normal conditions and calculated the adjusted *p*-value for each selected miRNA profile. We also reported miRNA tumour expression profiles based on the American Joint Committee on Cancer (AJCC) TNM staging. We grouped the clinical stages into four categories: (1) stage I; (2) stage II; (3) stage III; and (4) stage IV. We filtered out data with unassessed stage (stage x) or unreported information. Finally, we carried out a Spearman correlation analysis among the selected miRNA profiles and reported the associated *p*-values.

### 4.7. In Situ Hybridization

ISH was performed on formalin-fixed and paraffin-embedded tissue specimens surgically resected from patients diagnosed with BC. For each case analysed (a total of 22 breast tissues), we selected a block of tissue fixed in formalin and embedded in paraffin representative of the tumour and used it to obtain 4 μm-thick sections mounted on Superfrost Plus slides (Thermo Fisher Scientific, Waltham, MA, USA). The paraffin sections were placed in an oven at 60 °C for 45 min the day before ISH was performed and were stored overnight at 4 °C. Double digoxigenin (DIG)-labelled miRCURY LNA™ microRNA detection probes (Exiqon, Vedbæk, Denmark) were employed in this study. Specifically, we used 60 nM miR-145-5p target probe (Exiqon, Cat #619865-360), 60 nM miR-143-3p target probe (Exiqon, Cat #619872-360), 60 nM miR-125-5p target probe (Exiqon, Cat #611756-360), 40 nM scramble miR negative control probe (Exiqon, Cat #90-001) and 10 nM U6 positive control probe (Exiqon, Cat #90-002). The detection of microRNA by ISH was performed using the Enhanced miRCURY LNA microRNA ISH Optimization Kit (Exiqon, Vedbæk, Denmark) according to the manufacturer’s protocol. Briefly, formalin-fixed paraffin-embedded sections were deparaffinized in xylene, rehydrated through solutions of ethanol to phosphate-buffered saline (PBS, pH 7.4) and pre-treated by enzymatic digestion (Proteinase K at 20 μg/mL for 30 min at 37 °C). Hybridization was performed by adding 60 nM LNA detection probes diluted in Exiqon hybridization buffer (Exiqon, Vedbæk, Denmark, Cat #90000) to each slide and incubating for 1 h at 60 °C in a hybridizer oven. After hybridization, the sections were washed under stringent conditions. Stringent washes were performed in pre-heated saline-sodium citrate (SSC) buffers in 5-min washes at 60 °C: once in 5× SSC, twice in 1× SSC and twice in 0.2× SSC. Then, the slides were placed in 0.2× SSC at room temperature and washed in PBS with 0.1% Tween-20. The sections were blocked against the unspecific binding of the detection antibody using a blocking solution according to the manufacturer’s recommendations at room temperature for 15 min. The chromogenic detection of the miRNA LNA-ISH probe was performed with anti- anti-digoxygenin -alkaline phosphatase conjugate (1:800 dilution of the conjugate in blocking reagent, Roche Diagnostics GmbH, Mannheim, Germany) and incubated at room temperature for 1 h. Enzymatic development was performed by incubating the slides with 4-nitro-blue tetrazolium (NBT) and 5-bromo-4-chloro-3′-indolyl phosphate (BCIP) substrate (Roche) at 30 °C overnight to allow the formation of dark-blue 4-nitro-blue tetrazolium formazan precipitate. The following day, the sections were washed twice for 5 min in KTBT buffer (50 mM Tris–HCl, 150 mM NaCl, 10 mM KCl) and then shortly twice in water. Counterstaining using ISH nuclear fast red was performed for 1 min at room temperature. The sections were rinsed in tap water for 10 min, dehydrated through an increasing gradient of ethanol solutions and mounted with Eukitt mounting medium (VWR, Herlev, Denmark). A scrambled probe and U6 small nuclear RNA-specific probe were used as controls.

### 4.8. Measurement of CA 15-3

The CA15-3 serology test was performed in accordance with the manufacturer’s protocols and reference intervals. Specifically, this tumour marker was measured in 155 serum samples (*n* = 78 healthy donors and *n* = 77 BC patients) on a Dimension Vista® 1500 System (Siemens Healthcare Diagnostics Inc., Tarrytown, NY, USA) that uses the LOCI method (Siemens Healthcare Diagnostics, Eschborn, Germany), a homogeneous sandwich chemiluminescent immunoassay-based LOCI^®^ technology (Siemens Healthcare Diagnostics, Eschborn, Germany). The threshold value provided by the supplying company for CA15-3 was 35 UI/mL. For serum tumour marker determination, when its level exceeded a threshold value, the samples were considered to be positive; otherwise, they were considered to be negative.

### 4.9. Statistical Analysis

Statistical analyses were performed using the open source R Statistical Software. All *p*-values were two-sided, and *p* < 0.05 was considered statistically significant. We used Shapiro–Wilk analysis and the Levene test to assess the normality and homoscedasticity of the distribution of the data, respectively. We performed the Mann-Whitney non-parametric test to compare the expression of the selected miRNAs between the two groups (BC and healthy controls). Furthermore, stepwise logistic regression was implemented to assess the association between miRNA expression and the probability of pathology. ROC curves were constructed for each miRNA, CA15-3 and CEA, and AUC values with 95% confidence intervals were calculated to evaluate the predictive power of the selected molecules for detecting BC. We evaluated the diagnostic accuracy of each circulating miRNA, CA15-3 and CEA, and their combination. The non-parametric Spearman’s rank-order correlation and Mann-Whitney U test were performed to verify the correlations/associations among the biological markers and imaging parameters. To assess the association between the molecular and imaging parameters, the data were grouped according to positive or negative expression of ER, PR and HER2 receptors, low or high expression of Ki67 (low ≤20% and high >20%), grading, and staging as I, II, III, and IV, according to BC guidelines.

## 5. Conclusions

Our study highlighted that the addition of PET/MR biomarkers led to a better understanding of the relationships between circulating miRNAs and tumour biology in our BC population. Nevertheless, further studies with larger samples and a more homogeneous distribution of tumour subtypes and staging are needed to confirm our results.

## Figures and Tables

**Table 1:** Detailed clinicopathological characteristics of the study participants.

**Healthy Control Samples (*n* = 78)**	
Age range	28–84 years
Mean ± S.D.	51.05 ± 11.26
**Breast Cancer Samples (*n* = 77)**	
Age range	28–82 years
Mean ± S.D.	53.68 ± 12.02
**Subtype**	
Luminal A	16
Luminal B	50
HER2+	7
Triple negative	4
**Ki67**	
Low (1–20%)	21
High (21–100%)	56
**Grade**	
G1	1
G2	41
G3	35
**Stage**	
Stage I	1
Stage II	12
Stage III	42
Stage IV	22

S.D.: standard deviation; HER2+: Human epidermal growth factor receptor positive.

**Table 2:** Validation III. Logistic stepwise regression on the indicated molecules in a cohort of 78 CTR and 77 BC subjects. * Molecules that significantly (*p* < 0.05) estimate the probability of having a tumour.

Circulating Biomarkers	Estimate	Std. Error	*p*-Value
miR-125b-5p *	9.018	2.401	0.000248
miR-143-3p *	1.017 × 10^1^	2.644	0.000179
CA15-3 *	2.123 × 10^−3^	8.984 × 10^−4^	0.01941
CEA	4.866 × 10^−3^	7.095 × 10^−3^	0.4939

**Table 3:** Diagnostic accuracy of the indicated circulating biomarkers.

**miR-125b-5p**	**Disease +**	**Disease −**	**Total**	**Sensitivity**	**Specificity**	**Diagnostic** **Accuracy**
Test +	61 (TP)	16 (FP)	77	0.79 (CI 0.68–0.88)	0.79 (CI 0.69–0.88)	0.79 (CI 0.72–0.85)
Test −	16 (FN)	62 (TN)	78			
Total	77	78	155			
**miR-143-3p**	**Disease +**	**Disease −**	**Total**	**Sensitivity**	**Specificity**	**Diagnostic** **accuracy**
Test +	53 (TP)	20 (FP)	73	0.69 (CI 0.57–0.79)	0.74 (CI 0.63–0.84)	0.72 (CI 0.64–0.79)
Test −	24 (FN)	58 (TN)	82			
Total	77	78	155			
**CA15-3**	**Disease +**	**Disease −**	**Total**	**Sensitivity**	**Specificity**	**Diagnostic** **accuracy**
Test +	29 (TP)	1 (FP)	30	0.38 (CI 0.27–0.50)	0.99 (CI 0.93–1.00)	0.68 (CI 0.60–0.76)
Test −	47 (FN)	75 (TN)	122			
Total	76	76	152			
**CEA**	**Disease +**	**Disease −**	**Total**	**Sensitivity**	**Specificity**	**Diagnostic** **accuracy**
Test +	25 (TP)	8 (FP)	73	0.33 (CI 0.23–0.45)	0.89 (CI 0.80–0.95)	0.61 (CI 0.53–0.69)
Test −	51 (FN)	68 (TN)	82			
Total	76	76	152			

CI: confidence interval 95%; Disease +: BC patients, Disease −: healthy donors, TP: True Positive TP, FP: False Positive, FN: False Negative and TN: True Negative. CEA: carcinoembryonic antigen and BC: breast cancer.

**Table 4:** Correlation studies among the miRNAs selected and the imaging parameters extracted from tumour lesions. The table reports ρ and *p*-values (*p* < 0.05 was considered statistically significant).

	Stage II				Stage III		Stage IV	
	iAUC_mean_	Ki67	Kep_mean_	SUV_max_	iAUC_mean_	Ki67	Ki67	Ktrans_mean_
**miR-125-5p**	ns	ns	ns	ns	ns	ns	−0.513 *p*-value 0.029	−0.421 *p*-value 0.040
**miR-143-3p**	0.943 *p*-value 0.005	ns	0.943 *p*-value 0.005	0.829 *p*-value 0.042	0.938 *p*-value 0.044	ns	ns	ns

ns indicates non-significant results. iAUC_mean_: area under the concentration curve; SUV_max_: maximum standardized uptake value; Kep_mean_: mean reverse efflux volume transfer constant; Ktrans_mean_: mean forward volume transfer constantin.

## Supplementary Materials

The following are available online at https://www.mdpi.com/2072-6694/11/6/876/s1: Table S1: Statistical analyses, Table S2: Correlation analysis among the indicated miRNAs and hormonal receptor status of the lesions in a cohort of 77 BC patients, Table S3: Array Layout, Figure S1: Validation I: Relative expression of the selected miRNAs in a study population of 46 healthy donors (CTR) and 64 BC subjects, Figure S2: Expression analysis of miR-125b-5p, miR-143-3p and miR-145-5p by using the TCGA database.
