# Supplementary material for: Circulating miRNAs in Untreated Breast Cancer: An Exploratory Multimodality Morpho-Functional Study
Source: Cancers (Basel). 2019 Jun 22;11(6):876. doi: 10.3390/cancers11060876 (PMC6628327; doi:10.3390/cancers11060876)
Supplement: Supplementary file 1 [file cancers-11-00876-s001.pdf]

Supplementary materials

Table 1. Statistical analyses. (A) Logistic regression of the indicated miRNAs in a Study population of 46 healthy donors (CTR) and 64 BC subjects. * miRNA that significantly ($p < 0.05$) estimates the probability to have a tumor. (**B**) Logistic stepwise regression. * miRNA that significantly ($p < 0.05$) estimates the probability to have a tumor.

A

Logistic regression			
**miRNAs**	**Estimate**	**Std. Error**	***p*-value**
miR-125b-5p *	8.99381	3.91189	0.02350
miR-143-3p *	9.57245	4.51353	0.03631
miR-145b-5p	-1.47815	4.16086	0.72312
miR-100-5p *	-0.32904	5.40945	0.95161
miR-23a-3p	0.22855	0.42577	0.59257

B

Logistic stepwise regression			
**miRNAs**	**Estimate**	**Std. Error**	***p*-value**
miR-125b-5p *	9.72750	2.55870	0.000239
miR-143-3p *	8.46748	2.88744	0.004113

Table S2. Correlation analysis among the indicated miRNAs and hormonal receptor status of the lesions in a cohort of 77 BC patients. $p < 0.05$ was considered statistically significant.

	**miR-125b-5p**	**miR-143-3p**	**miR-145-5p**
Estrogen Receptor	p-value = 0.8322	p-value = 0.4848	p-value = 0.648
Progesteron Receptor	p-value = 0.6328	p-value = 0.4121	p-value = 0.4218
HER2	p-value = 0.6856	p-value = 0.7519	p-value = 0.6828
Lesion size	p-value = 0.8288	p-value = 0.5154	p-value = 0.8559

HER2: Human epidermal growth factor receptor

Table 3. Array Layout.

**hsa-let-7a-5p A01**	**hsa-miR-1 A02**	**hsa-miR-100-5p A03**	**hsa-miR-106b-5p A04**	**hsa-miR-10b-5p A05**	**hsa-miR-122-5p A06**	**hsa-miR-124-3p A07**	**hsa-miR-125b-5p A08**	**hsa-miR-126-3p A09**	**hsa-miR-133a A10**	**hsa-miR-133b A11**	**hsa-miR-134 A12**
hsa-miR-141-3p B01	hsa-miR-143-3p B02	hsa-miR-146a-5p B03	hsa-miR-150-5p B04	hsa-miR-155-5p B05	hsa-miR-17-5p B06	hsa-miR-17-3p B07	hsa-miR-18a-5p B08	hsa-miR-192-5p B09	hsa-miR-195-5p B10	hsa-miR-196a-5p B11	hsa-miR-19a-3p B12
hsa-miR-19b-3p C01	hsa-miR-200a-3p C02	hsa-miR-200b-3p C03	hsa-miR-200c-3p C04	hsa-miR-203a C05	hsa-miR-205-5p C06	hsa-miR-208a C07	hsa-miR-20a-5p C08	hsa-miR-21-5p C09	hsa-miR-210 C10	hsa-miR-214-3p C11	hsa-miR-215 C12
hsa-miR-221-3p D01	hsa-miR-222-3p D02	hsa-miR-223-3p D03	hsa-miR-224-5p D04	hsa-miR-23a-3p D05	hsa-miR-25-3p D06	hsa-miR-27a-3p D07	hsa-miR-296-5p D08	hsa-miR-29a-3p D09	hsa-miR-30d-5p D10	hsa-miR-34a-5p D11	hsa-miR-375 D12
hsa-miR-423-5p E01	hsa-miR-499a-5p E02	hsa-miR-574-3p E03	hsa-miR-885-5p E04	hsa-miR-9-5p E05	hsa-miR-92a-3p E06	hsa-miR-93-5p E07	hsa-let-7c E08	hsa-miR-107 E09	hsa-miR-10a-5p E10	hsa-miR-128 E11	hsa-miR-130b-3p E12
hsa-miR-145-5p F01	hsa-miR-148a-3p F02	hsa-miR-15a-5p F03	hsa-miR-184 F04	hsa-miR-193a-5p F05	hsa-miR-204-5p F06	hsa-miR-206 F07	hsa-miR-211-5p F08	hsa-miR-26b-5p F09	hsa-miR-30e-5p F10	hsa-miR-372 F11	hsa-miR-373-3p F12
hsa-miR-374a-5p G01	hsa-miR-376c-3p G02	hsa-miR-7-5p G03	hsa-miR-96-5p G04	hsa-miR-103a-3p G05	hsa-miR-15b-5p G06	hsa-miR-16-5p G07	hsa-miR-191-5p G08	hsa-miR-22-3p G09	hsa-miR-24-3p G10	hsa-miR-26a-5p G11	hsa-miR-31-5p G12
cel-miR-39-3p H01	cel-miR-39-3p H02	SNORD61 H03	SNORD68 H04	SNORD72 H05	SNORD95 H06	SNORD96A H07	RNU6-2 H08	miRTC H09	miRTC H10	PPC H11	PPC H12

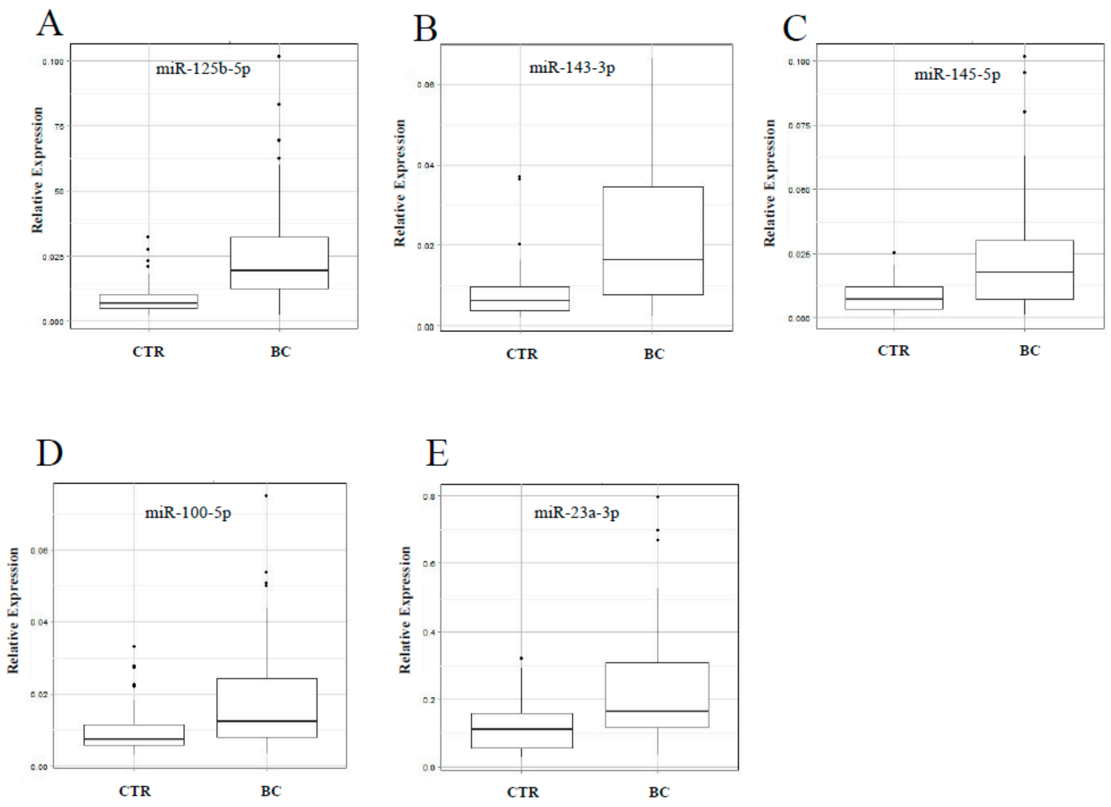

F

miRNAs	p-value	Median CTR N= 46	25th-75th percentiles	Median BC N=64	25th-75th percentiles	Fold change
miR-125b-5p	1.15e-10	0.0069	0.00265-0.02703	0.0195	0.00669-0.7517	2.8
miR-143-3p	4.33e-07	0.0062	0.00257-0.03437	0.0163	0.00369-0.05931	2.6
miR-145-5p	1.62e-05	0.0073	0.00127-0.02074	0.0177	0.00241-0.08666	2.3
miR-100-5p	0.0005	0.0073	0.00321-0.02754	0.0122	0.00381-0.05199	1.7
miR-23a-3p	3.58e-05	0.1107	0.034664-0.29245	0.1632	0.05059-0.68104	1.47

Figure S1. Validation I: Relative expression of the selected miRNAs in a study population of 46 healthy donors (CTR) and 64 BC subjects. Box plot analyses performed to show the relative expression of circulating (**A**) miR-125b-5p, (**B**) miR-143-3p, (**C**) miR-145-5p, (**D**) miR-100-5p and (**E**) miR-23a-3p. (**F**) Statistical analyses. Table includes: p-value; median value; 25th-75th percentiles; and fold change of each miRNAs up-regulated in plasma of BC patients vs CTR.

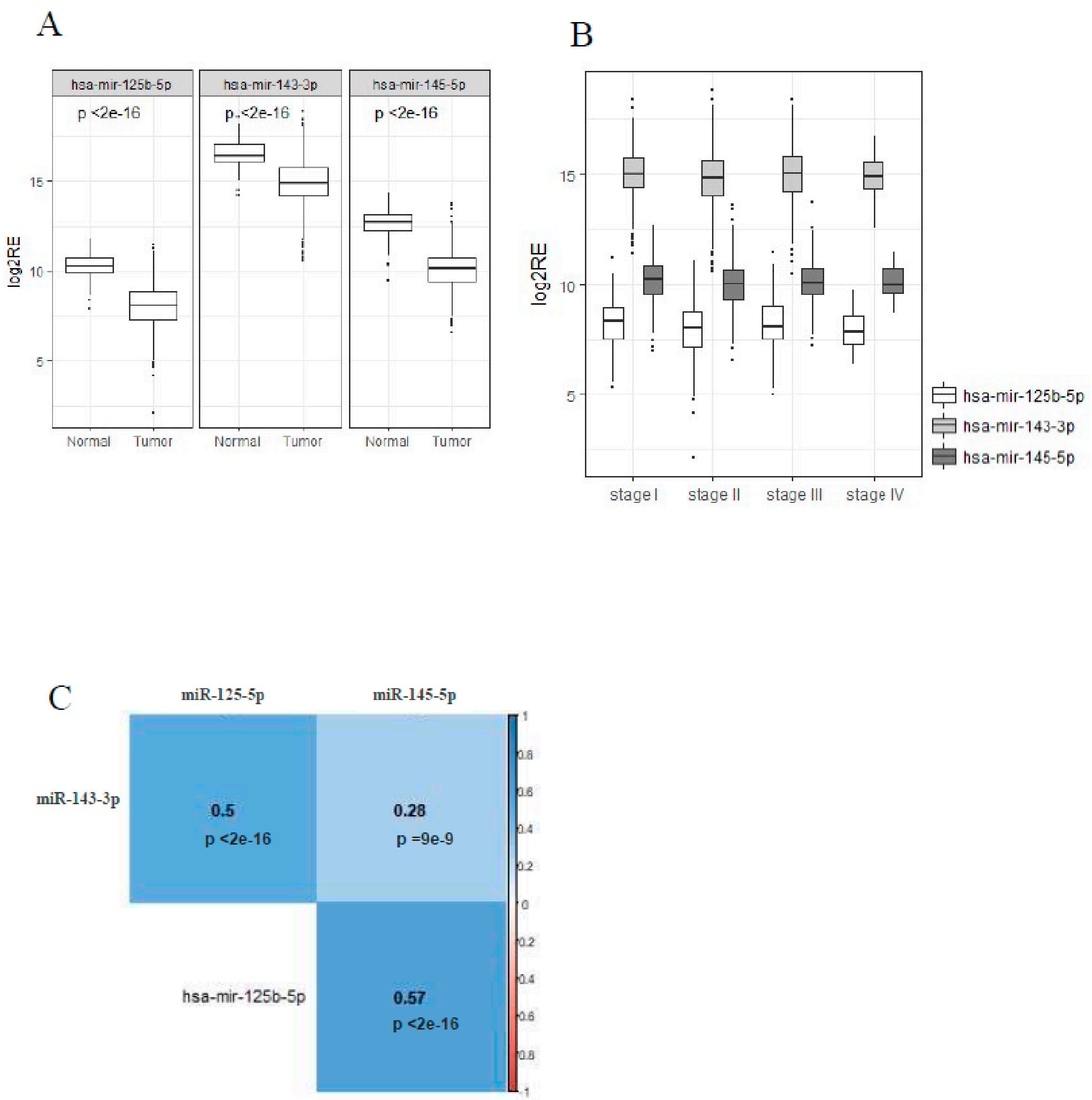

Figure S2. Expression analysis of miR-125b-5p, miR-143-3p and miR-145-5p by using the The Cancer Genome Atlas database (TCGA). Expression levels of the indicated miRNAs in breast tissue samples (normal = 104, and tumor = 1076) (**A**), trend of miRNAs profile with staging of the disease (**B**), and among them (**C**).
